# Bacteriophages: The viruses for all seasons of molecular biology

**DOI:** 10.1186/1743-422X-2-19

**Published:** 2005-03-15

**Authors:** Jim D Karam

**Affiliations:** 1 Department of Biochemistry, Tulane University Health Sciences Center, New Orleans, Louisiana USA

## Abstract

Bacteriophage research continues to break new ground in our understanding of the basic molecular mechanisms of gene action and biological structure. The abundance of bacteriophages in nature and the diversity of their genomes are two reasons why phage research brims with excitement. The pages of *Virology Journal *will reflect the excitement of the "New Phage Biology."

## 

The launching of *Virology Journal *comes at a time of resurgence of interest in the basic biology of the bacteriophages and the impact that these viruses have on earth's ecology, evolution of microbial diversity and the control of infectious disease. Since playing an important part in the birth of Molecular Biology more than 50 years ago [1], phage research has continually broken new ground in our understanding of the basic molecular mechanisms of gene action and biological structure [2]. This trend shows no signs of waning. In a recent international meeting entitled The New Phage Biology [3], the program was largely devoted to emerging frontiers of research that have been empowered by a rapid accumulation of genome sequence information from a wide variety of bacteriophages. Phage genomics is revealing novel biochemical mechanisms for replication, maintenance and expression of the genetic material and is providing new insights into origins of infectious disease and the potential use of phage gene products and even whole phage as therapeutic agents.

Two reasons why the new era of phage research brims with excitement are the abundance of bacteriophages in nature and the diversity of their genomes. Phage is probably the most widely distributed biological entity in the biosphere, with an estimated population of >10^30 ^or ~10 million per cubic centimeter of any environmental niche where bacteria or archaea reside [4]. At one level, there is diversity in the types of phages that infect individual or interrelated bacterial species. At another level, there is diversity among genomically related phages that do not share the same bacterial hosts. One example is the lytic Enterobacterial dsDNA phage T4, which has relatives that are specific to *Aeromonas*, *Vibrio*, *Acinetobacter*, marine and other bacterial species. The genomes of a few T4-like phages have been sequenced and found to indeed share homologies with T4, but to also differ from one another in size, organization of the T4-like genes and content of other putative genes and DNA mobile elements (). It appears that phage families like the T4-related phages have learned to cross bacterial species barriers and possess plastic genomes that can acquire and lose genetic cassettes through their travels in the microbial world. In essence, genomes of the dsDNA phages may be repositories of the genetic diversity of all microorganisms in nature.

In addition to evolving by serving as traffickers of microbial genes, phage genomes evolve through the accumulation of mutations in both acquired and core genes. Sequence divergence among homologues of the essential genes for phage propagation within a phage family can be used as a source of information about the determinants of specificity of the protein-protein and protein-nucleic-acid interactions that underlie biological function. Phages are excellent sources of many enzymes and biochemical transactions that are broadly represented in all divisions of life. The large numbers of phylogenetic variants of biologically interesting proteins and nucleic acids that one can derive from sequenced phage genomes are treasure troves for studies of biological structure in relation to function. Interest in phage and phage gene products as potential therapeutic agents is also increasing rapidly and is likely to have profound impact on the pharmaceutical industry and biotechnology in general over the coming years. There is a general sense that the best is yet to come out of phage research.

## Conclusion

We anticipate that the pages of *Virology Journal *will reflect the excitement of the "New Phage Biology" by publishing reports in the areas of Ecology and Taxonomy, Genomics and Molecular Evolution, Regulation of Gene Expression, Genome Replication and Maintenance, Protein and Nucleic Acid Structure, Virus assembly, Biotechnology, Pathogenesis, Therapeutics and more. It would be especially interesting to see submissions of phage genome sequence briefs and their biological implications.

## Competing interests

The author(s) declare that they have no competing interests.
